# Deciphering the effects of PYCR1 on cell function and its associated mechanism in hepatocellular carcinoma

**DOI:** 10.7150/ijbs.58026

**Published:** 2021-06-01

**Authors:** Yanzhen Xu, Wenpu Zuo, Xiao Wang, Qinle Zhang, Xiang Gan, Ning Tan, Wenxian Jia, Jiayi Liu, Zhouquan Li, Bo Zhou, Dong Zhao, Zhibin Xie, Yanjun Tan, Shengfeng Zheng, Chengwu Liu, Hongtao Li, Zhijian Chen, Xiaoli Yang, Zhaoquan Huang

**Affiliations:** 1 Department of pathology, Affiliated hospital of Guilin Medical University, Guilin, 541001, Guangxi, China; 2 Scientific Research Center, Guilin Medical University, Guilin, 541001, Guangxi, China; 3 Guangxi Health Commission Key Laboratory of Disease Proteomics Research, Guilin Medical University, Guilin, 541001, Guangxi, China; 4 Department of Pathology, Affiliated Hangzhou First People's Hospital, School of Medicine, Zhejiang University, 310000, Hangzhou, China; 5 Department of Pathology, the First Affiliated Hospital of Guangxi Medical University, Nanning, 530000, Guangxi, China.; 6 Medical Scientific Research Center, Guangxi Medical University, Nanning, 530000, Guangxi, China.; 7 Genetic and metabolic central laboratory, the maternal and children's health hospital of Guangxi, Nanning, 530000, Guangxi, China.; 8 Department of Urology, the Five Affiliated Hospital of Guangxi Medical University, Nanning, 530000, Guangxi, China.; 9 Department of Pathophysiology, Guangxi Medical University, Nanning, 530000, Guangxi, China.; 10 Department of Clinical Laboratory, the First Affiliated Hospital of Guangxi Medical University, Nanning, 530000, Guangxi, China.

**Keywords:** Pyrroline-5-carboxylate reductase 1, Hepatocellular carcinoma, Antitumor, Antimetastasis, Molecular docking, RNA-seq, miRNA

## Abstract

Overexpression of pyrroline-5-carboxylate reductase 1 (PYCR1) has been associated with the development of certain cancers; however, no studies have specifically examined the role of PYCR1 in hepatocellular carcinoma (HCC). Based on The Cancer Genome Atlas expression array and meta-analysis conducted using the Gene Expression Omnibus database, we determined that *PYCR1* was upregulated in HCC compared to adjacent nontumor tissues (P < 0.05). These data were verified using quantitative real-time polymerase chain reaction, western blotting, and immunohistochemistry analysis. Additionally, patients with low PYCR1 expression showed a higher overall survival rate than patients with high *PYCR1* expression. Furthermore, PYCR1 overexpression was associated with the female sex, higher levels of alpha-fetoprotein, advanced clinical stages (III and IV), and a younger age (< 45 years old). Silencing of *PYCR1* inhibited cell proliferation, invasive migration, epithelial-mesenchymal transition, and metastatic properties in HCC *in vitro* and *in vivo.* Using RNA sequencing and bioinformatics tools for data-dependent network analysis, we found binary relationships among PYCR1 and its interacting proteins in defined pathway modules. These findings indicated that PYCR1 played a multifunctional role in coordinating a variety of biological pathways involved in cell communication, cell proliferation and growth, cell migration, a mitogen-activated protein kinase cascade, ion binding, *etc*. The structural characteristics of key pathway components and PYCR1-interacting proteins were evaluated by molecular docking, and hotspot analysis showed that better affinities between PYCR1 and its interacting molecules were associated with the presence of arginine in the binding site. Finally, a candidate regulatory microRNA, miR-2355-5p, for *PYCR1* mRNA was discovered in HCC. Overall, our study suggests that PYCR1 plays a vital role in HCC pathogenesis and may potentially serve as a molecular target for HCC treatment.

## Introduction

Pyrroline-5-carboxylate reductase 1 (PYCR1), the most abundant isoform in the PYCR family, is an enzyme that catalyzes the NAD(P)H-dependent conversion of Δ^1^-pyrroline-5-carboxylate (P5C) to proline 1. Members of the human PYCR family (PYCR1-3) and proline dehydrogenase 1 (PRODH) have an established metabolic relationship, known as the proline-P5C cycle 2. PRODH oxidizes proline to P5C, whereas proteins from the PYCR family reduce P5C back to proline 3. The proline-P5C cycle plays a pivotal role in a myriad of cellular processes, including amino acid metabolism, and is involved in the maintenance of the intracellular redox potential and mitochondrial integrity 4. The important regulatory contribution of PYCR1 is the catalysis of P5C to proline. Recent studies have also reported that PYCR1 converts Δ^1^-piperideine-6-carboxylate into pipecolic acid and is associated with the expression of matrix metalloproteinases 1, 4. Furthermore, PYCR1 has demonstrated an ability to protect cells from mitochondrial fragmentation during oxidative stress 4-6. Meanwhile, mutations in *PYCR1* have been shown to induce the development of cutis laxa, a multisystem disorder characterized by premature aging, the appearance of wrinkled and lax skin, joint laxity, and a general developmental delay 7.

Hepatocellular carcinoma (HCC) is the third leading cause of cancer-related mortality worldwide and is responsible for approximately 700,000 deaths annually 8, 9. Many risk factors are associated with the development of HCC, among which the most critical factors include chronic hepatitis C or hepatitis B infection, alcoholic cirrhosis, nonalcoholic steatohepatitis, and exposure to aflatoxin B1 10-12. Given the asymptomatic nature of HCC in the early stages, majority of HCC cases are not detected until reaching advanced stages, which results in incurable disease states 13, 14. Moreover, patients who are diagnosed with advanced HCC are not candidates for definitive-intent therapies, such as resection, transplantation, or ablation 15. Although first-line therapy with sorafenib is considered the standard of care for patients with advanced HCC, outcomes remain poor. As a result, the general prognosis is poor, with a 5-year overall survival (OS) rate of 3-5% 16, 17. Nevertheless, recent advances have been made in the treatment of HCC, including sophisticated locoregional therapy and its associated assisted technology, newly available drugs and procedures associated with transcatheter arterial therapy, and biomarker-matched molecular-targeted therapy. Gene therapy, including gene-targeted therapy, is one of the most promising therapeutic options for HCC.

Overexpression of PYCR1 has been reported in many cancers, including non-small cell lung cancer, prostate cancer (PCa), colon cancer, and breast cancer 18-22. Knockdown of PYCR1 was found to significantly inhibit PCa cell growth and colony formation 1, whereas PYCR1 overexpression has been correlated with poor prognoses in patients with specific cancers 1, 18. PYCR1 expression has also been significantly associated with breast cancer tumor size, grade, and invasiveness. It was reported that *PYCR1* silencing could inhibit the expression of insulin receptor substrate 1 (IRS1) and insulin resistance via the suppression of the c‑Jun N‑terminal kinase (JNK) signaling pathway, which subsequently inhibited HCC cell proliferation and promoted cell apoptosis 23. However, it remains unclear whether PYCR1 is involved in other biological pathways related to HCC pathogenesis and development.

In the present study, we aimed to analyze the relationship between PYCR1 silencing and HCC *in vitro and in vivo*, in order to provide a better understanding of the mechanisms underlying HCC cell growth and survival. Furthermore, RNA sequencing (RNA-seq), bioinformatics analyses, and molecular docking were used to elucidate protein interactions that are associated with the function of PYCR1. The results will probably provide us a potential drug target for therapy or a biomarker for diagnosis and prognosis. These results will help us better understand the role of PYCR1 in the pathogenesis and development of HCC.

## Methods

### Cells and reagents

The HCC cell lines used in this study included Huh7 cells and LM3 cells. Because the PYCR1 expression of these two cells were higher than the other cells (Figure S1). The HCC cell lines were obtained from the China Center for Type Culture Collection (Wuhan, China). These cell lines were cultured in Dulbecco's modified Eagle's medium (DMEM; Gibco, USA) supplemented with 10% fetal bovine serum (FBS; Gibco) and maintained in a humidified incubator with 5% CO_2_ at 37 °C.

The following antibodies were used in immunoblotting and immunofluorescence studies: anti-β-actin (HRP-60008; Proteintech Group, China), anti-PYCR1 (131081; Proteintech Group), anti-E-cadherin (PA5-19479; ThermoFisher Scientific, USA), anti-β-catenin (ab32572; Abcam, USA), anti-N-cadherin (ab76011; Abcam), and anti-vimentin (ab92547; Abcam).

Reagents and materials used for the analysis of cell proliferation, migration, and invasion and for RNA-seq included Cell Counting Kit‑8 (CCK‑8; CK04, Dojindo Molecular Technologies, Inc., Rockville, MD, USA), Transwell plates (8 μm pore size; Corning, Inc.), RNA Nano 6000 assay kit (Agilent Technologies, CA, USA), and NEB Next® Ultra™ RNA library prep kit for Illumina® (#E7530L; New England Biolabs, USA).

### Specimen collection

A total of 106 tumor samples and adjacent nontumor liver tissues were obtained from patients who had been pathologically diagnosed with HCC and undergone a partial hepatectomy at The Affiliated Hospital of the Guilin Medical University. None of the patients received adjuvant chemotherapy or radiotherapy prior to surgery, and all cases were independently confirmed by two senior pathologists. The clinicopathological information for all cases was retrieved from the hospital's clinical database. This study was approved by the ethics committee of the Guilin Medical University, and informed consent was obtained from the patients.

### Meta-analysis

Seven microarray datasets (GSE84598, GSE57957, GSE39791, GSE31370, GSE36411, GSE89377, and GSE87630) were downloaded from the Gene Expression Omnibus database (http://www.ncbi.nlm.nih.gov/geo/). Multiple probes were used for the detection of the *PYCR1* gene, and the mean expression value was calculated for each probe 24. Expression data for *PYCR1* mRNA in HCC tissues were extracted from the datasets using the R software, and then a meta-analysis was performed. Standardized mean differences and 95% confidence intervals were calculated for pooled values and indicated expression differences. Cochran's Q and I^2^ tests were used to evaluate heterogeneity. Based on the level of heterogeneity, two models were applied to the overall meta-analysis. A random-effect model was chosen when there was significant heterogeneity among all samples (P < 0.05, I^2^ > 50%), and a fixed-effect model was used when there was no significant heterogeneity, as indicated by statistical analysis 25-28.

### Data mining using the TCGA database

Clinical and RNA-seq data for TCGA cohorts were downloaded from the Xena Public Data Hubs (https://xena-browser.net/). Gene expression was quantified experimentally using the Illumina HiSeq 2000 RNA-seq platform. Three TCGA datasets including gene expression RNA-seq (Illumina HiSeq, n=423) dataset, phenotype (n=438) dataset, and miRNA expression RNA-seq (Illumina Hiseq, n=420) dataset were used in the study, which belongs to TCGA Liver Cancer cohort. A total of 413 samples, including 363 HCC tissues and 50 adjacent non-HCC tissues were selected for this study. Detailed patient information and *PYCR1* expression levels are summarized in Table 1. The TCGA database was also employed to confirm the potential OS and relapse-free survival of patients, as well as receiver operating characteristic (ROC) signatures of PYCR1 29.

### Prediction of miRNAs

Four frequently used online tools, including miRNet (https://www.mirnet.ca), miRDB (http://www.mirdb.org), miRTarBase (http://mirtarbase.mbc.nctu.edu.tw/php/index.php), and TargetScan (http://www.targetscan.org/vert_72/) were used to predict the upstream regulatory miRNAs of *PYCR1*. Only miRNAs appearing in two or more sets of the results were chosen as candidate miRNAs.

### Reverse transcription-polymerase chain reaction

Total RNA was extracted using TRIzol reagent. Reverse transcription was carried out in a 20 µL reaction volume with 1 µg total RNA using the PrimeScript RT reagent kit (No. RR036A; TaKaRa, Japan) according to the manufacturer's instructions. Quantitative real-time polymerase chain reaction (qRT-PCR) was performed using a SYBR Green master mix (Roche, Switzerland) with a StepOne sequence detection system (Applied Biosystems). The qRT-PCR cycling conditions were as follows: initial denaturation at 95 °C for 10 min, followed by 40 cycles of denaturation at 95 °C for 15 s, annealing at 58-60 °C for 60 s, and extension at 72 °C for 10 s. The mRNA expression level was quantified using the 2^-ΔΔCt^ method and normalized to that of β-actin used as an endogenous control 30. All primers are listed in Table S1.

### Western blot analysis

Proteins were extracted using RIPA lysis buffer (Solarbio Science and Technology, Beijing, China) containing a protease inhibitor cocktail (Beyotime, Shanghai, China). Protein quantification was performed using a bicinchoninic acid protein assay kit (Leagene Biotechnology, Beijing, China). Proteins were separated on 10% sodium dodecyl sulfate-polyacrylamide gels and transferred onto polyvinylidene difluoride membranes. The membranes were blocked with 5% skim milk and then incubated with primary antibodies, including β-actin (1:8,000), PYCR1 (1:1,000), E-cadherin (1:1,000), β-catenin (1:5,000), N-cadherin (1:2,000), and vimentin (1:3,000), at 4 ℃ overnight. After being washed with Tris-buffered saline with Tween 20 for four times, the membranes were incubated with a horseradish peroxidase-conjugated secondary antibody at room temperature for 1 h. Immunoreactive bands were visualized using the BeyoECL Plus kit (Beyotime).

### Immunohistochemistry

Formalin-fixed and paraffin-embedded 4 μm-thick tissue sections were deparaffinized, rehydrated, and heated with citric acid buffer for antigen retrieval for 15 min. The samples were then placed in 0.3% hydrogen peroxide for 30 min to block endogenous peroxidase activity and treated with blocking serum for 20 min. The slides were incubated with an anti-PYCR1 primary antibody (GTX114693; GeneTex, USA) at a 1:900 dilution at 4 ℃ overnight, followed by incubation with a biotinylated secondary antibody for 25 min. The negative control slides were incubated with PBS only. The slides were rinsed in phosphate-buffered saline (PBS, pH 7.2), then counterstained with hematoxylin, dehydrated, and mounted in a neutral resin. The staining intensities and the proportions of positive cells in all tissues were analyzed and scored by two experienced pathologists. The results were analyzed using immunoreactivity scores based on the staining intensity and the proportion of stained cells. The staining intensity was scored as follows: 0 = unstained; 1 = weakly stained; 2 = moderately stained; and 3 = strongly stained. The proportion of stained cells was scored as follows: 0 = negative; 1 = 1-10%; 2 = 11-50%; 3 = 51-80%; and 4 > 80%. The intensity score was multiplied by the proportion of staining score to obtain an immunoreactivity score. A total score greater than 3 was considered positive expression; that of 1 to 3 was considered low expression; and 0 was considered negative expression.

### RNA interference of PYCR1 by lentivirus

Downregulation of *PYCR1* was achieved using the following three interfering RNAs (RNAis): RNAi 1, 5'-gaGGGTCTTCACCCACTCCTA-3'; RNAi 2, 5'-tgAGAAGAAGCTGTCAGCGTT-3'; and RNAi 3, 5'-caCAGTTTCTGCTCTCAGGAA-3'. The RNAis were obtained from Shanghai GeneChemCo and were inserted between the *Age*I and *Eco*RI sites of the GV248 vector. The short hairpin RNAs (shRNA) of GV-NC-shRNA and GV-PYCR1-shRNA were constructed as control lentivirus and shPYCR1 lentivirus for further experiment. To establish stable HCC cell lines with silenced *PYCR1*, 1.5 μg/mL puromycin was added to the culture media 48 h after the initiation of virus infection, and the expression of the green fluorescent protein (GFP) was observed. Cells were then trypsinized and cultured in a 100 cm^2^ dish to allow for expansion. The silencing of *PYCR1* was confirmed by qRT-PCR and immunoblotting assays. Empty vectors were used as controls.

### Cell proliferation assay

Cell proliferation was assessed using the CCK-8 solution according to the manufacturer's instructions**.** Briefly, Huh7 cells (1.5 × 10^3^) from the shNC and shPYCR1 groups were seeded into a 96-well plate. After 24, 48, 72, 96, and 120 h of treatment, 10 μL of the CCK-8 solution was added to each well, and incubation continued for 2 h. The absorbance was measured at 450 nm using a microplate reader (BioTek Instruments, Inc., VT, USA). Each experiment was performed in triplicate.

### Scratch wound assay

Huh7 and LM3 cells were seeded in a serum-free DMEM into 6-well plates (7-8 × 10^6^ cells/well) and incubated for 24 h to reach 80% confluence. A sterile 10 μL pipette tip was then used to gently and slowly scratch the cell monolayer. Images of the wound were captured in 10 random fields using a light microscope (Olympus, Japan) at a 100× magnification. The gap width in the *PYCR1*-silenced cell groups was compared to that in the control using the Photoshop software at different time points. Each experiment was repeated three times.

### Cell invasion assay

The *in vitro* invasion assay was conducted using 24-well Transwell plates with Matrigel (BD Biosciences, Franklin Lakes, NJ, USA). Cells (1 × 10^4^) of Huh7 and LM3 were suspended in 200 µL of a serum-free DMEM, seeded into the upper Transwell chamber, and cultured at 37 °C for 24 h, while 600 µL of DMEM containing 10% FBS was simultaneously added to the lower chamber. The invaded cells were fixed with methanol, stained with a 2.5% crystal violet stain solution (Solarbio), and then observed and photographed using an optical microscope. Five visual fields were randomly selected for quantification in each group.

### Flow cytometry

Allophycocyanin-conjugated annexin V and 7-aminoactinomycin D (BD Biosciences, San Jose, CA, USA) were used to analyze the cell apoptosis rate. Huh7-shNC and Huh7-shPYCR1 cells were washed twice with PBS and treated with 0.25% pancreatin (without EDTA) to facilitate digestion. After adjusting the cell density to 1 × 10^5^/mL, the cells were incubated with fluorescent antibodies at room temperature for 25 min, then pelleted, and resuspended. The samples were analyzed using a flow cytometer (BD Biosciences) and the FlowJo software.

### *In vivo* tumor formation

Male BALB/c nude mice (6-7 weeks old) were obtained from the Shanghai Institute of Materia Medica (Chinese Academy of Sciences, Shanghai, China). Cells stably transfected with the shPYCR1 or shNC (1.5 × 10^7^ cells in 100 µL of DMEM) were injected subcutaneously into the upper left flank region of the mice. Tumor diameters were measured using digital calipers every 4 days. After 4 weeks of observation, the mice were sacrificed under anesthesia, and xenograft tissues were collected. All animal procedures were performed in accordance with the protocol approved by the Guilin Medical University Experimental Animal Center.

### RNA sequencing

RNA purity was assessed using a K5500 spectrophotometer (Kaiao, Beijing, China), and RNA integrity and concentration were measured using the RNA Nano 6000 assay kit for the Bioanalyzer 2100 system (Agilent Technologies). A total of 2 μg of RNA from each sample was used as an input material for the RNA sample preparation. Sequencing libraries were generated using the NEB Next® Ultra™ RNA library prep kit for Illumina® according to the manufacturer's instructions. After library analysis, RNA-seq was completed using the Illumina HiSeq X Ten sequencing platform, and paired-end sequence reads were obtained.

Quality control of the raw sequencing data was performed using the FastQC tool v0.11.9 ( https://www.bioinformatics.babraham.ac.uk/). Clean RNA-seq reads were aligned to the GRCh38 human genome using HISAT v2.1.0 with default settings 31. The aligned files were then processed using Samtools v1.9 32, and FeatureCounts v1.6.3 was used to quantify the number of reads aligned to the coding regions of the genome 33. Finally, DESeq2 v1.22.2 was used with the R/Bioconductor package to normalize read counts and identify differentially expressed genes (DEGs) 34.

### Bioinformatics analysis

To analyze the functional roles of the DEGs, Gene Ontology and Kyoto Encyclopedia of Genes and Genomes pathway analyses were conducted. For proteins with multiple functions, those that are most common were assigned. Protein-protein interaction (PPI) networks were established using the STRING database.

### Molecular docking

The structures of PYCR1 and its interacting proteins were downloaded from the RCSB Protein Data Bank (PDB) database (https://www.rcsb.org/). The preparation of the PDB files, including the removal of extraneous water molecules, addition of hydrogen molecules, and adjustment of pH-sensitive protonation, was performed using the SYBYL-X 2.0 software. Protein-protein docking was performed using the docking program HEX 8.0.0. The KFC2 (Knowledge-based FADE and Contacts) server was used to calculate protein-protein binding interface hotspots, which are defined as small fractions of residues accounting for a large fraction of the binding affinity 35. Positive controls, interacting proteins with PYCR1 (experiment confirmed), were determined through literature review 36-40. The average docking energy among positive control-PYCR1 complexes (-1460.178 kcal·mol^-1^) was calculated to assess the docking profile in our study. Further details were recorded in Table S2.

### Statistical analysis

All data were analyzed using the SPSS 17.0 software (SPSS, Chicago, IL, USA). Data are presented as the mean ± standard deviation. One-way analysis of variance was used to compare differences between multiple groups, and Student's *t*-test was used to analyze differences between two groups. Bivariate correlations between study variables were calculated using a chi-squared test or Fisher's exact test. Survival analyses were conducted and visualized using the Kaplan-Meier method. The diagnostic ability was evaluated using an ROC curve. The sensitivity and specificity were obtained using an optimal cutoff with the max Youden index. P < 0.05 was considered statistically significant.

## Results

### Meta-analysis of PYCR1 expression in HCC datasets

**Figure 1:**
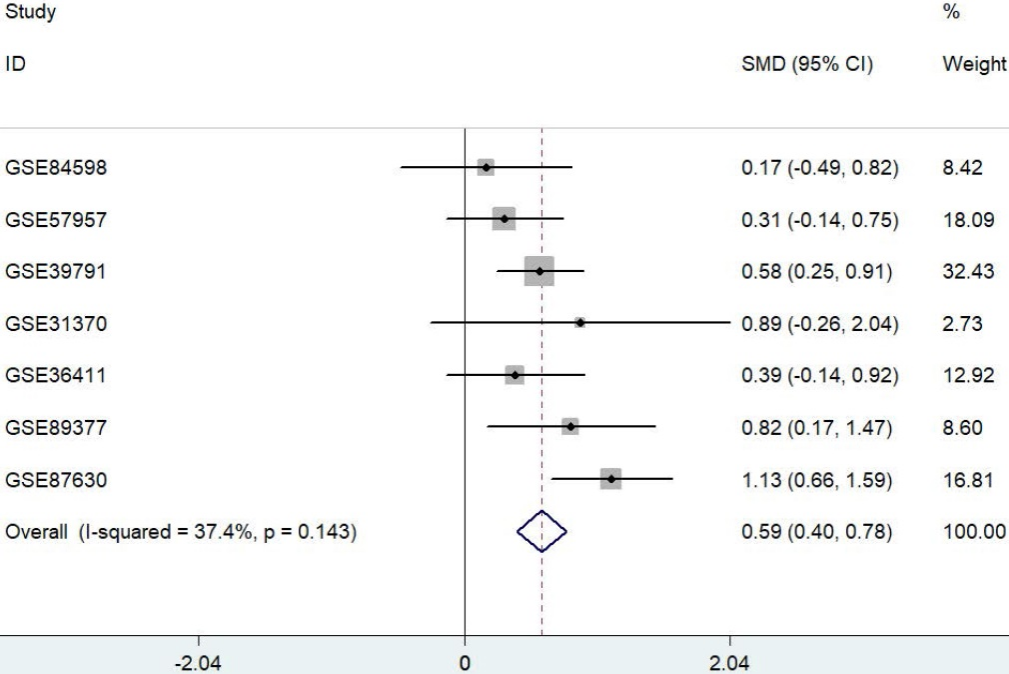
** Forest plot depicting the association between PYCR1 mRNA expression and HCC development using a fixed-effect model.** SMD: standard mean difference (tumor vs. adjacent tissue). All data were selected from GEO database.

Data were extracted from seven published and publicly available HCC datasets. Only datasets containing both HCC and their adjacent nontumor samples were used for further analysis. A meta-analysis approach was applied to these datasets as outlined in Figure 1. The results showed that PYCR1 expression was a risk factor for HCC.

### Data mining of the TCGA dataset

**Figure 2:**
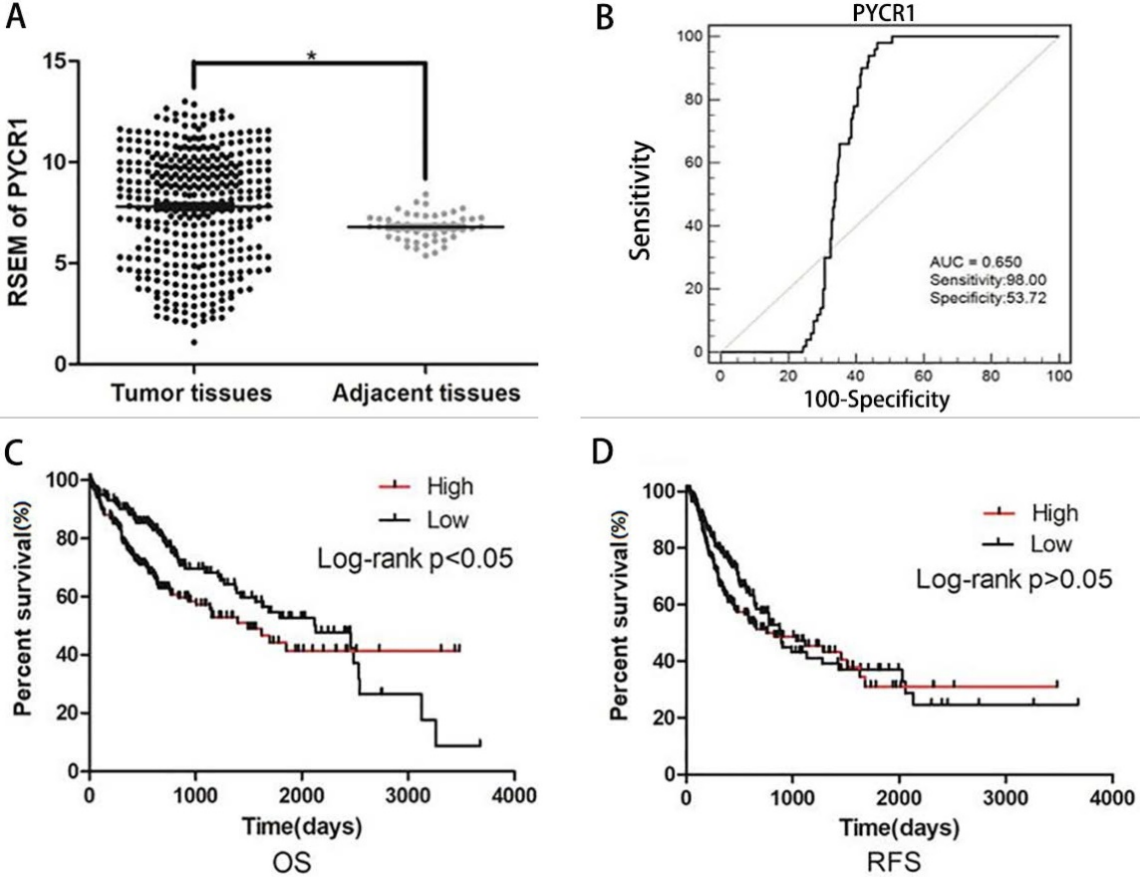
**PYCR1 data mining in TCGA datasets**. (A) RSEM of PYCR1. Significant differences were observed between tumor tissues and adjacent tissues. Student's* t*-tests were used to analyze the differences between two groups. (B) ROC curve for PYCR1 (**P < 0.01). (C) Overall survival (OS) between patients with high and low PYCR1 expression levels. Higher PYCR1 expression was associated with poorer OS (P < 0.05) (Kaplan-Meier analysis). (D) Recurrence-free survival (RFS) between patients with high and low PYCR1 (Kaplan-Meier analysis).

The expression of *PYCR1* mRNA was analyzed in 413 samples, including 363 HCC tissues and 50 adjacent nontumor tissues, from the TCGA dataset. Increased *PYCR1* expression was found in the HCC tissues compared with the adjacent nontumor tissues (Figure 2A). Furthermore, the correlation between clinicopathological features and *PYCR1* expression was investigated in 363 tumor samples. The results indicated that elevated *PYCR1* expression was correlated with higher alpha-fetoprotein (AFP) levels, higher clinical staging (stages III and IV), and a younger age (< 45 years old) (*p* < 0.05; Table 1). In addition, females were shown to have higher levels of *PYCR1* than males (*P* < 0.05; Table 1).

We also performed ROC curve and Kaplan-Meier curve analyses on these TCGA samples. The area under the ROC curve was determined to be 0.650 (Figure 2B). Furthermore, as shown in Figure 2C, patients with low *PYCR1* expression exhibited a higher OS rate than patients with high *PYCR1* expression (*P* < 0.05). However, recurrence-free survival indicated that there was no difference in the prognosis between high and low *PYCR1*-expressing patients (*P* > 0.05; Figure 2D).

### Verification of PYCR1 expression in HCC tissues and relationship between PYCR1 expression and HCC clinicopathological features

**Figure 3:**
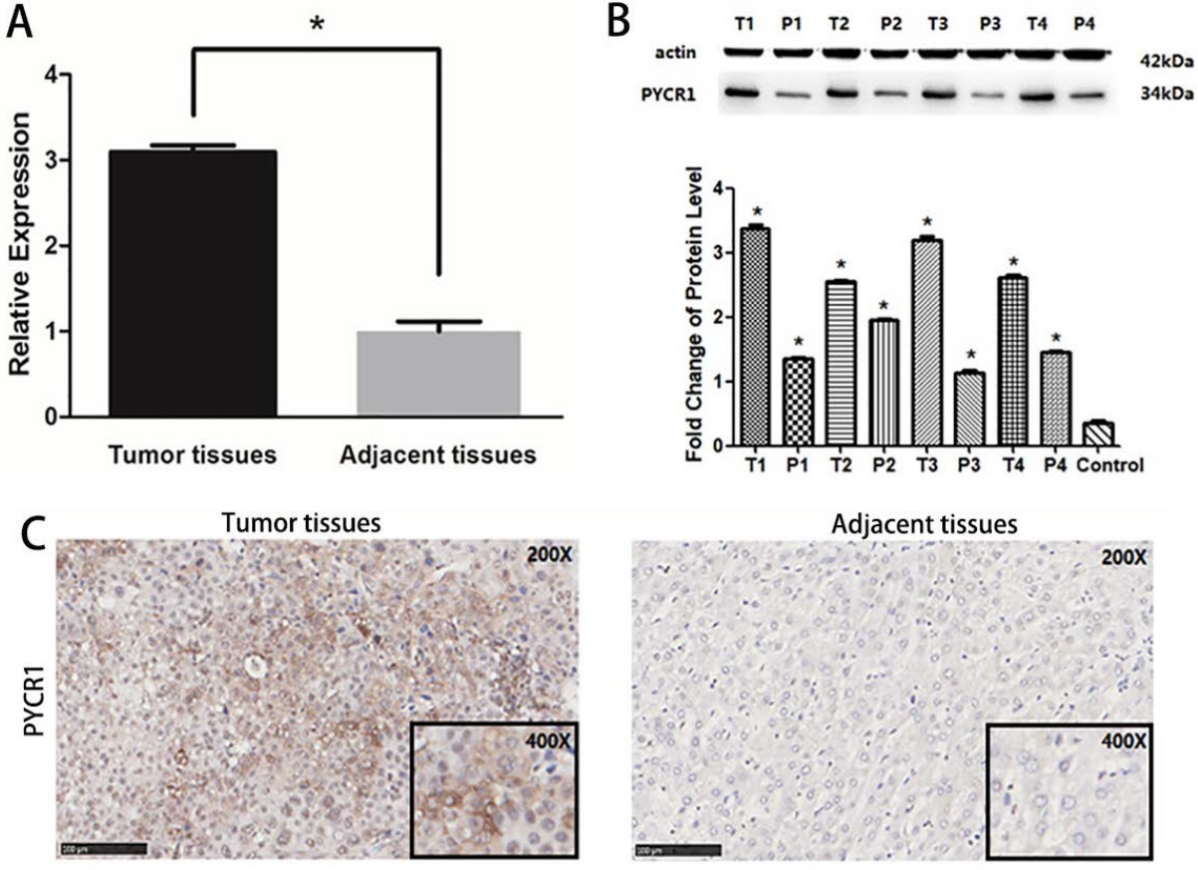
** Expression of PYCR1 in human HCC tissues.** (A) Western blot analysis of PYCR1. Lanes 1, 3, 5, and 7: cancer tissues; lanes 2, 4, 6, and 8: adjacent tissues. (B) Relative mRNA expression of PYCR1. Tissues are listed on the X-axis, and the relative expression level is shown in the Y-axis. T: tumor; P: adjacent tissues. (C) Representative images showing immunohistochemical staining of PYCR1 in tumor tissues and adjacent tissues.

*PYCR1* mRNA expression was found to be significantly higher in tumor tissues than in adjacent nontumor tissues in 106 paired tumor and adjacent tissues sets (*P* < 0.05; Figure 3A). Further, the PYCR1 protein expression was consistent with that of mRNA (Figure 3B and 3C). Higher expression of PYCR1 was also associated with a younger age (< 45 years old) and higher clinical staging (stages III and IV). However, no association was detected between PYCR1 expression and other examined clinical and demographic features, including sex, tobacco smoking, alcohol consumption, hepatitis B surface antigen levels, AFP levels, alanine aminotransferase (ALT) levels, and tumor size (Table 2).

### Downregulation of PYCR1 inhibits cell proliferation, migration, invasion, and tumor growth

**Figure 4:**
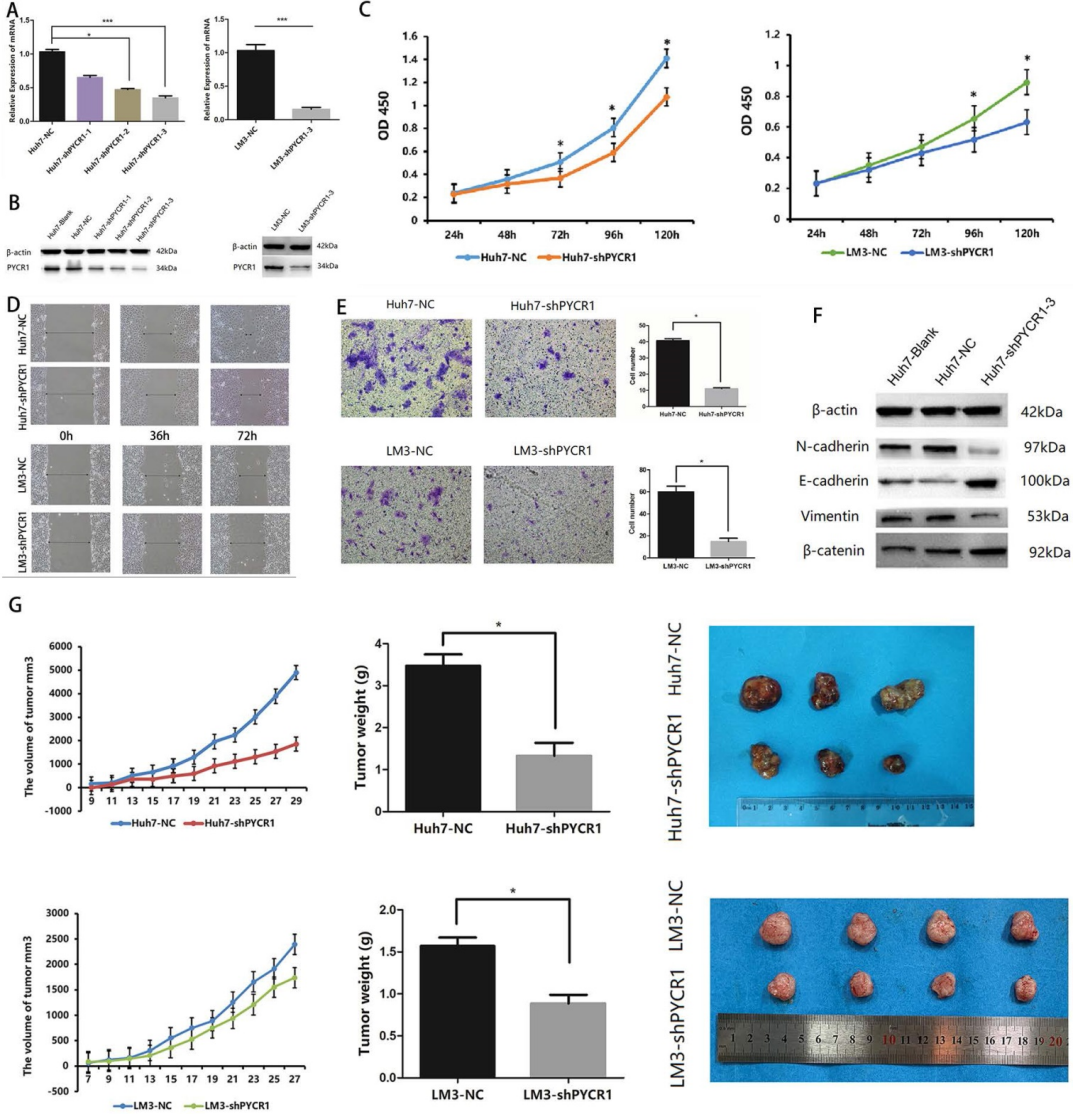
** Knockdown of PYCR1 inhibits proliferation, migration, invasion, EMT and tumor growth capacities of HCC cells.** (A-B) Western blot analysis and qRT-PCR were used to detect PYCR1 expression in Huh7 and LM3 cell lines transfected with three kinds of PYCR1 RNAi. (C) The CCK8 analysis revealed down-regulation of PYCR1 caused inhibition of cellular proliferation. (D) Scratch-wound assay showed that down-regulation of PYCR1 in Huh7 cells inhibited cell migration. (E) Down-regulation of PYCR1 reduced the invasiveness of HCC cells (data are shown as averages ± SD, n = 5, *P < 0.05). (F) Western blot analysis of EMT-related proteins showing the expression of E-cadherin, N-cadherin, vimentin, and β-catenin as quantified by western blot. (G) Down-regulation of PYCR1 in Huh7 cells inhibited tumor growth in nude mice (*P < 0.05).

To assess the effects of PYCR1 in HCC, we established PYCR1 interference by infecting with control lentivirus and shPYCR1 lentivirus in HCC cells. We detected the PYCR1 expression in HCC cell lines by Western blot. And we found the PYCR1 expression of Huh7 was higher than others in non-metastatic cell lines. Additionally, PYCR1 expression in LM3 cells was the highest among three high-metastatic cell lines. Taken together, Huh7 and LM3 cell lines, which have comparably high expressions of PYCR1 among non-metastatic and metastatic cells respectively, were chosen to conduct further target gene silencing experiments (Figure S1). Compared with controls, infected with shPYCR1 lentivirus led to suppressed PYCR1 expression in both mRNA and protein levels. The transcription of PYCR1 was suppressed dramatically by shPYCR1(1-3) compared with shNC in Huh7 (Figure 4A and 4B). Significantly, we found shPYCR1(3) could more efficiently suppress the expression of PYCR1 than others. And shPYCR1(3) obviously suppressed the expression of PYCR1 mRNA and protein in LM3 (Figure 4A and 4B).

Downregulation of PYCR1 expression was found to inhibit cell proliferation (Figure 4C). The migration capacity and invasiveness were assessed using Transwell plates and a wound scratch assay. As shown in Figure 4D and 4E, downregulation of PYCR1 expression significantly inhibited the migration and invasiveness of Huh7 and LM3 cells* in vitro*. However, no significant differences were observed in the levels of apoptosis between the experimental and control groups (data not shown). Furthermore, we quantified the expression levels of proteins associated with epithelial-mesenchymal transition (EMT) and determined whether their expression was affected by downregulation of PYCR1. Our results suggested that the expression of E-cadherin and β-catenin was significantly increased in cells with downregulated PYCR1 expression, whereas that of N-cadherin and vimentin was decreased (Figure 4F).

We further analyzed the role of PYCR1 *in vivo* using an immunodeficient nude mouse model. Compared with the control mice, those with downregulated PYCR1 expression exhibited a delayed onset of tumor growth and a smaller tumor volume after 4 weeks of observation (Figure 4G).

### Global profiling of differentially expressed mRNAs

**Figure 5:**
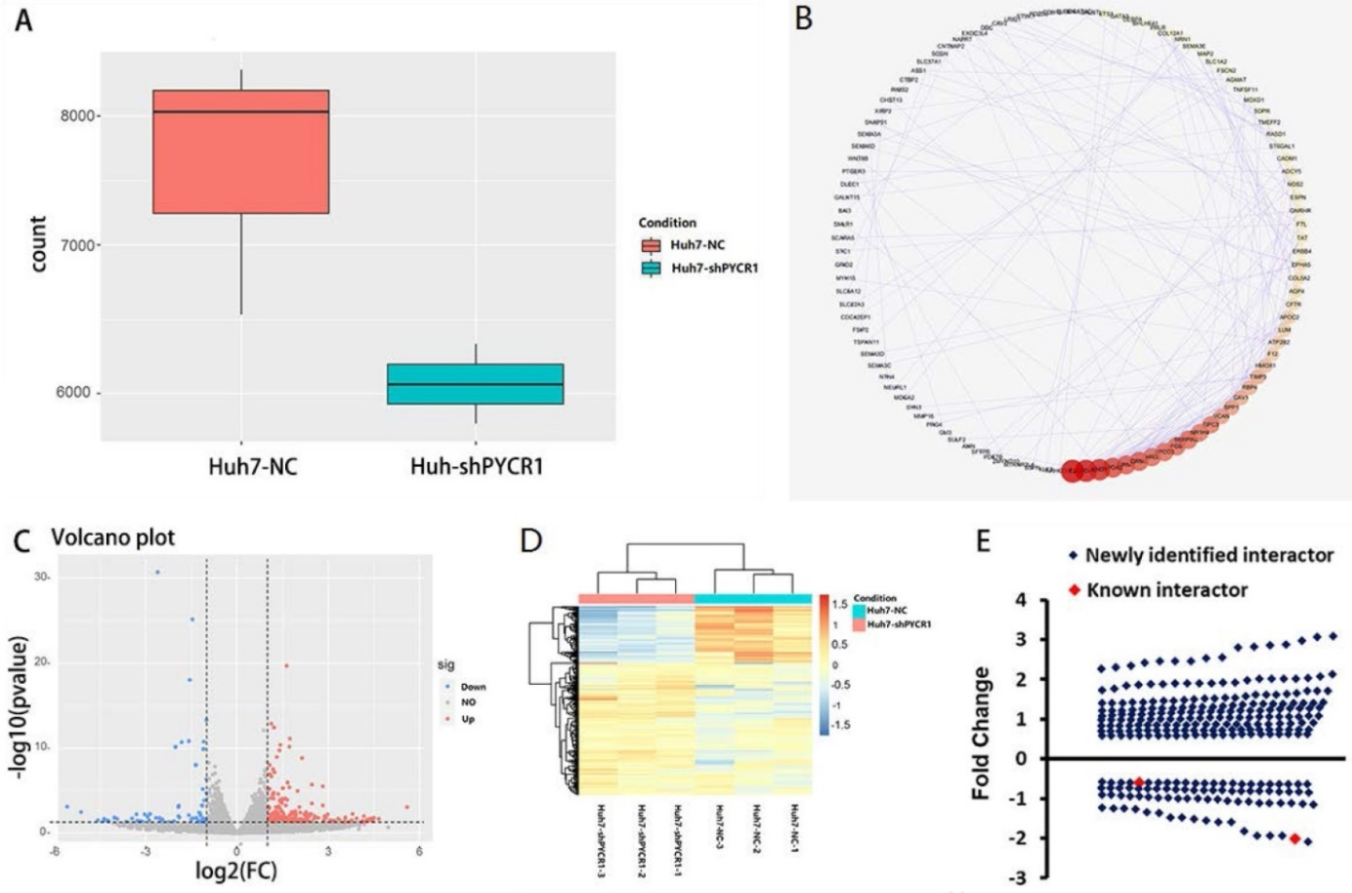
** Down-regulation of PYCR1 resulted in differential mRNA expression, as demonstrated by RNA-seq analysis.** (A) The PYCR1 mRNA expression level between PYCR1-silenced Huh7 cells and Huh7 cell control cells as determined by RNA-seq analysis. The gene counts were obtained by quantifying the number of reads that aligned with PYCR1 using the feature Counts programs. (B) The protein-protein interaction networks associated with the differentially expressed genes from RNA-Seq analysis. The interaction network is presented as a Cytoscape degree sorted circle summary layout; the nodes are shown as circles and the edges are shown as lines linking two nodes. (C) Volcano plot. Blue spots: up-regulated genes; red spots: down-regulated genes. (D) Heatmap. Significantly differentially expressed genes observed between control Huh7 cells and PYCR1-silenced Huh7 cells. (E) Distribution of up/down-regulated genes after the silencing of PYCR1 in HCC cells. Red spots: known genes; blue spots: newly identified genes.

Global mRNA profiling was performed in Huh7 cells with either normal or downregulated expression levels of *PYCR1*. Genes were categorized as up- or downregulated when their expression fold changes were > 1.5 or < 0.67 compared to the control group, respectively (*P* < 0.05). Based on these parameters, 569 genes were found. After removing noncoding genes, 425 DEGs were identified, of which 298 were upregulated and 127 were downregulated following *PYCR1* silencing (Figure 5A-D). Some of these genes encoded proteins that were known to interact with PYCR1; however, most of the genes were identified for the first time (Figure 5E).

### Data-dependent network analysis of PYCR1-interacting proteins in HCC cells

**Figure 6:**
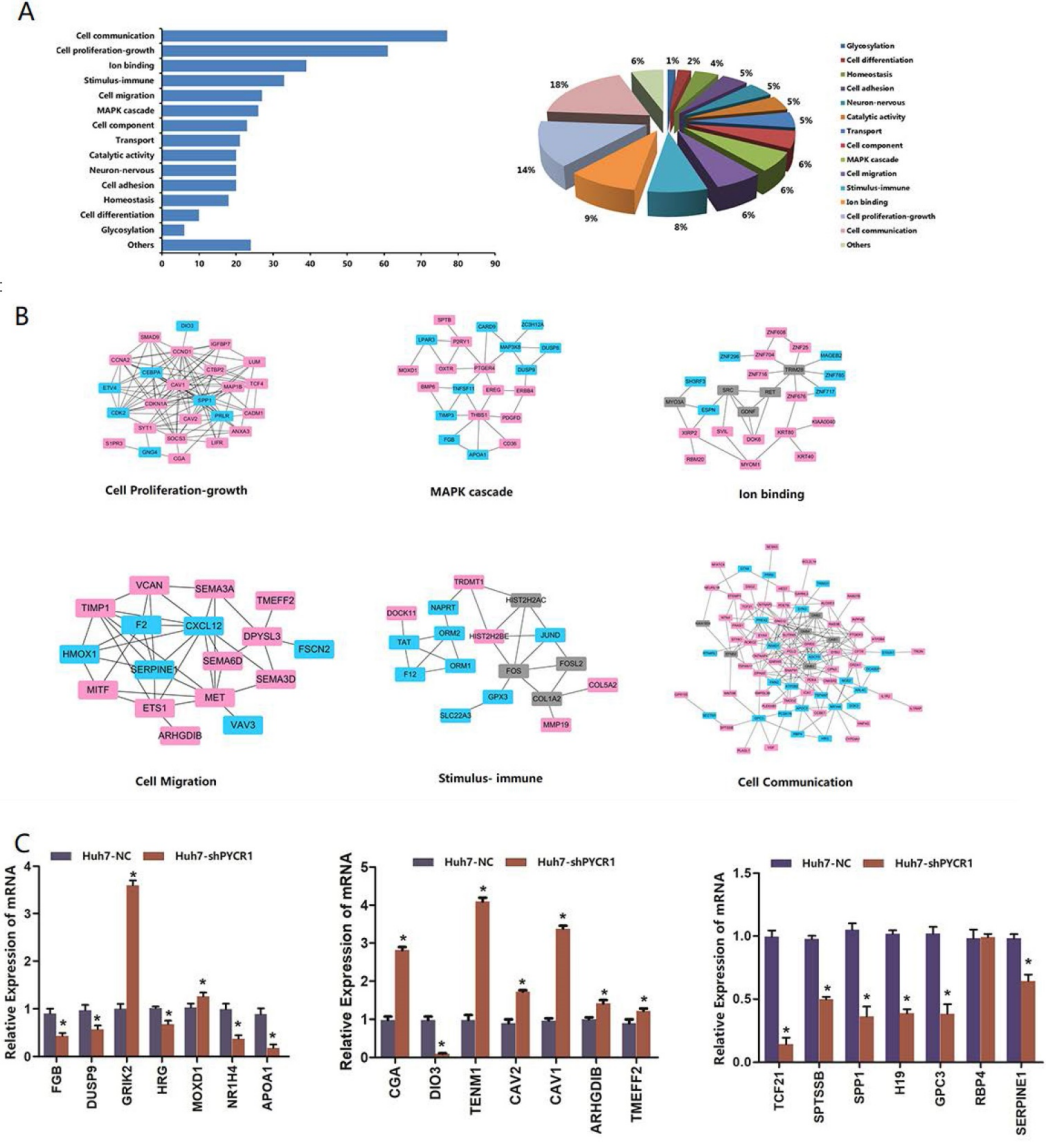
** Biological functional groups of differentially expressed genes in HCC cells.** (A) Multiple functional categories. (B) Pathway modules. Red spot: up-regulated genes after silencing of PYCR1; blue spot: down-regulated genes after silencing of PYCR1; gray spot: potential node genes. (C) Validation of mRNA levels of differentially expressed genes obtained via RNA-seq with qRT-PCR.

As shown in Figure 6A**,** each DEG was assigned to a specific functional category, including cell communication, cell proliferation and growth, cell migration, a mitogen-activated protein kinase (MAPK) cascade, ion binding, immune stimulus, cell component, transport, and catalytic activity. PPI analysis further demonstrated that many of the proteins were associated with each other (Figure 6B).

### Validation of the HCC-specific PYCR1 interactome dataset

In our selected pool of PYCR1-interacting proteins, more attention was paid to the genes known to be involved in vital pathway modules, such as cell communication, cell proliferation and growth, cell migration, an MAPK cascade, and ion binding. Based on their large fold changes and biological functional analysis, some of the DEGs were further confirmed by qRT-PCR, and the results were consistent with those of RNA-seq (Figure 6C).

### Molecular docking

**Figure 7:**
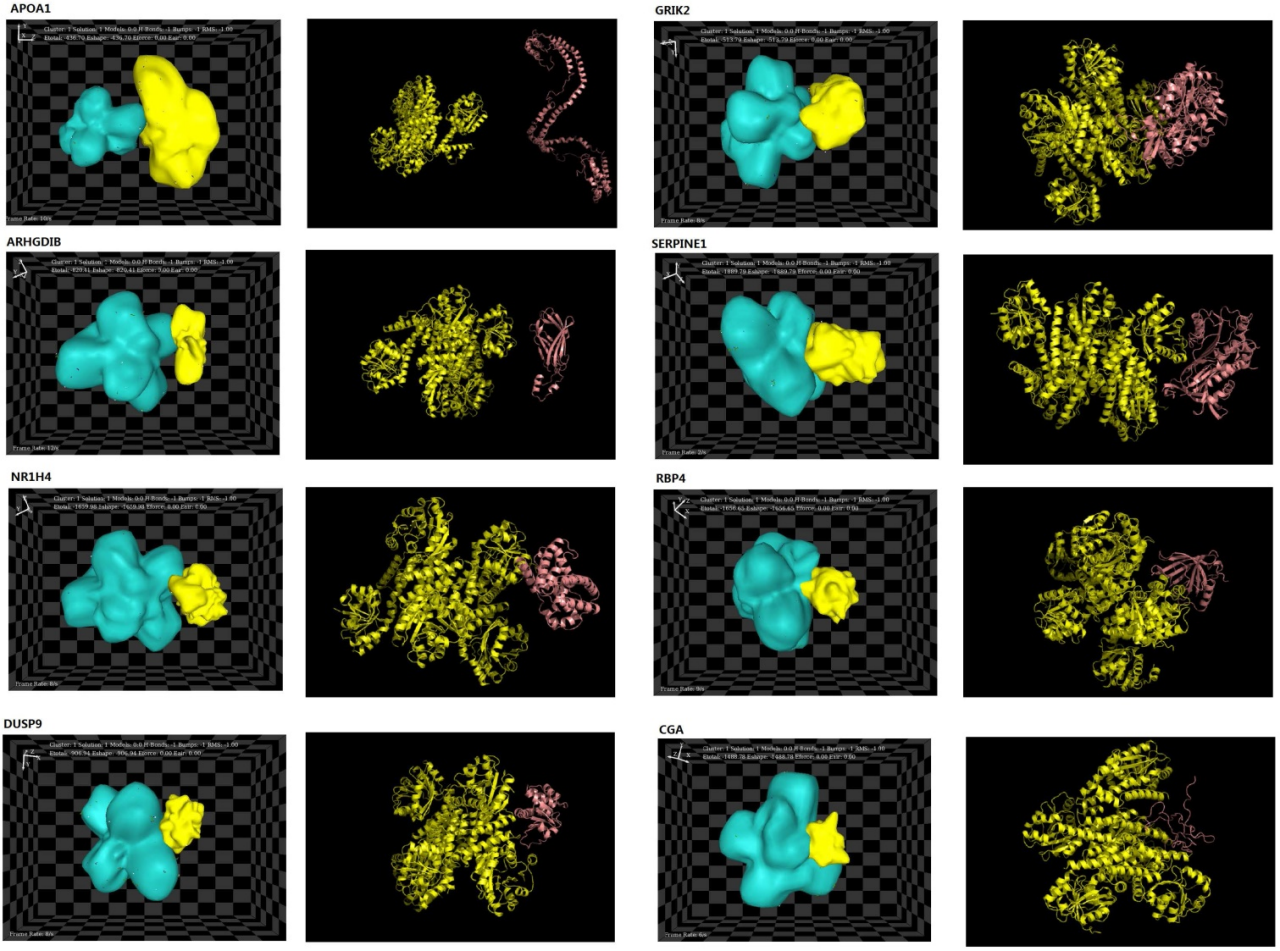
** Docking results of PYCR1 and its target proteins including APOP1, GRIK2, ARHGDIB, DUSP9, CGA, RBP4, NR1H4, and SERPINE1.** PDB files for each target protein and PYCR1 were collected from https://www.rcsb.org/.

The structures of eight interacting proteins, which were associated with key pathway modules, such as MAPK pathways, cell migration, cell communication, and cell proliferation/growth, and identified downstream of PYCR1, were found in the PDB database. The information obtained from the PDB files and HEX docking results is shown in Table 3. Total energy values (E_total_) for PYCR1 and its eight target proteins were determined to be as follows: apoptogenic protein 1 (APOP1; -436.7 kcal·mol^-1^), glutamate ionotropic receptor kainite type subunit 2 (GRIK2; -513.79 kcal·mol^-1^), RhoGD12 (ARHGDIB; -820.41 kcal·mol^-1^), dual specificity phosphatase 9 (DUSP9; -906.94 kcal·mol^-1^), chorionic gonadotropin subunit alpha (CGA; -1,488.78 kcal·mol^-1^), retinol-binding protein 4 (RBP4; -1,656.65 kcal·mol^-1^), nuclear receptor subfamily 1 group H member 4 (NR1H4; -1,659.98 kcal·mol^-1^), and serpin family E member 1 (SERPINE1; -1,889.79 kcal·mol^-1^). The complexes generated during docking are shown in Figure 7, and the E_total_ values reflect the possibility of binding, with a lower value correlating with easier and stronger binding to other proteins 35. The results of the hotspot analysis are summarized in Figure 7. We found that better affinities between PYCR1 and its interacting molecules were associated with the presence of arginine (Arg) in the binding site. The diagnostic value of PYCR1, combined with its interacting proteins, was further tested based on the RNA-seq data from TCGA by binary logistic regression (Figure S2 and Table S3).

### miR-2355-5p might be an upstream regulator for PYCR1 mRNA in HCC

**Figure 8:**
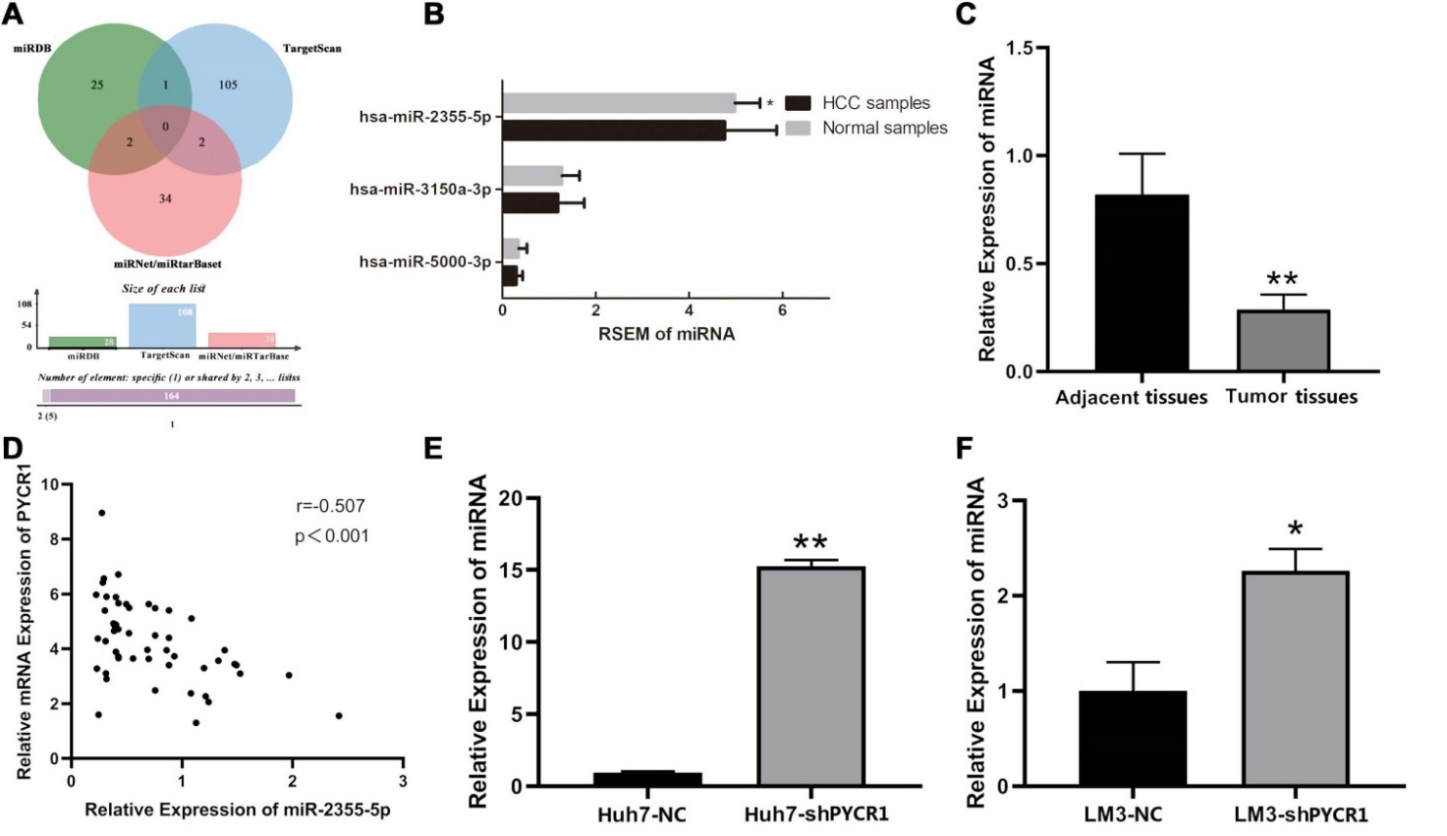
** Potential miRNAs targeting PYCR1 mRNA in HCC.** (A) Online prediction of upstream regulatory miRNAs of PYCR1 mRNA; (B) Expression levels of miR-2355-5p, miR-3150a-3p, and miR-5000-3p in HCC and adjacent samples from TCGA; (C) Correlation analysis between PYCR1 mRNA and miR-2355-5p; (D) Validation of qRT-PCR: decreased miR-2355-5p in HCC tissues compared to adjacent tissues; (E, F) Up-regulated miR-2355-5p in HCC cell lines of Huh7 and LM3 after silencing PYCR1.

All predicted miRNAs from the four miRNA databases are listed in Table S4. According to the quantitative Venn diagram (Figure 8A), five candidates, including miR-1253, miR-6081, miR-3150a-3p, miR-2355-5p, and miR-5000-3p, were determined to potentially target *PYCR1* mRNA. Based on the TCGA RNA-seq data, only miR-2355-5p was differentially expressed between HCC tissues and adjacent nontumor tissues and was downregulated in HCC samples compared to adjacent nontumor samples (Figure 8B). The expression levels of miR-3150a-3p and miR-5000-3p were similar in HCC and adjacent nontumor samples, while no data pertaining to miR-1253 and miR-6081 were available in the TCGA database. The validation using qRT-PCR showed that miR-2355-5p expression was decreased in HCC tissues compared to adjacent nontumor tissues (Figure 8C), with a negative correlation between *PYCR1* mRNA and miR-2355-5p expression (Figure 8D). After silencing *PYCR1*, miR-2355-5p was found to be upregulated in HCC cell lines (Figure 8E and 8F).

## Discussion

PYCR1 plays an important role in the normal physiological functions of cells and in the development of various diseases 41, 42. In particular, previous studies have shown that PYCR1 is closely associated with the development of various cancers, including PCa, lymphoma, and breast cancer 1, 18, 43. However, the specific mechanism responsible for the role of PYCR1 in cancer development and progression has not been adequately elucidated. Our findings suggest that PYCR1 is involved in HCC pathogenesis and may thus serve as a diagnostic and therapeutic target for HCC management.

In the present study, *PYCR1* mRNA and protein expression levels were found to be higher in HCC tissues than in adjacent nontumor tissues. Analysis of *PYCR1* expression in patients from the TCGA cohort revealed that the upregulation of *PYCR1* correlated with sex, elevated AFP levels, higher clinical staging (stages III and IV), and a younger age (< 45 years old). Furthermore, *PYCR1* upregulation was found to be associated with a poor OS. These results suggest that PYCR1 expression may be an effective predictive factor for OS in patients with HCC. Similar correlations between PYCR1 expression and clinical features (age and clinical staging) were also observed in our patients with HCC; however, there was no association between PYCR1 expression and AFP level or sex. This discrepancy may be due to a largely Caucasian population in the TCGA database, whereas our HCC tissues were exclusively obtained from Chinese patients. Thus, ethnic diversity may impact the effect of PYCR1 on HCC development. Additional HCC samples from patients of different ethnicities should be included in further studies.

A previous study has shown that silencing of *PYCR1* induces apoptosis and cell cycle arrest 1; however, we did not obtain similar results. We employed lentivirus-mediated shRNAs to silence the expression of *PYCR1* in Huh7 and LM3 HCC cells and to determine the effect of PYCR1 on various cellular functions. The results revealed that silencing *PYCR1* induced a marked inhibition of cell proliferation, migration, and invasion. Further, the results of the HCC xenograft mouse study indicated that silencing *PYCR1* significantly inhibited the tumor growth.

EMT has been shown to play an important role in tumorigenesis by inhibiting epithelial function and upregulating the expression of mesenchymal-related genes to enhance invasiveness and metastasis 44. Moreover, epithelial cell characteristics and cell-cell adhesion were found to be disrupted, whereas migration and invasion properties were improved by EMT 45. Specific markers of EMT (E-cadherin, N-cadherin, vimentin, and β-catenin) were detected in our study. Cadherins are important factors in maintaining the stability of cell-cell adherens junctions and cellular signal transduction 44, 45. Additionally, the expression of E-cadherin is decreased and that of N-cadherin is increased in epithelial cells that are converted into fusoid mesenchymal cells during EMT. Furthermore, as one of the primary cytoskeletal components in mesenchymal cells, vimentin, whose concentration has been shown to increase during EMT, is closely associated with the differentiation, metastasis, and invasion of cancer cells 46. β-catenin is a multifunctional protein, which, together with E-cadherin, serves a structural role in adherens junctions, thereby contributing to sustained unrestricted proliferation, survival, and metastatic spread of cancer cells 47. In our study, the silencing of *PYCR1* caused increased expression of E-cadherin and β-catenin and decreased expression of N-cadherin and vimentin. These results suggest that PYCR1 promotes HCC migration and invasiveness by inducing EMT.

To better understand the downstream molecular events involved in the effects of PYCR1 on HCC invasiveness and metastasis, we performed RNA-seq to obtain DEG profiles following *PYCR1* silencing. Two specific genes, namely, secreted phosphoprotein 1 (*SPP1*) and C-X-C motif chemokine 12 (*CXCL12*) were identified to be strongly associated with PYCR1. Previous studies have reported that PYCR1 can activate SPP1 to regulate cell adhesion 48, 49. Furthermore, PYCR1 expression is regulated by the CXCL12⁄SDF-1/CXCR7 signaling pathway, which regulates the expression of genes involved in cell cycle control, amino acid metabolism, and ligase activity 50, 51. The other DEGs that were identified in our study did not exhibit any obvious synergistic or antagonistic functions with PYCR1, and thus, their mechanisms in relation to PYCR1 require further elucidation.

The results of our functional analysis showed that the identified DEGs were primarily enriched in 14 functional categories, including cell communication, MAPK cascade, and cell migration. Cell communication is crucial for morphogenesis, cell differentiation, homeostasis, growth, and cell-cell interaction 52. Downregulated histidine-rich glycoprotein (HRG), NR1H4, and transcription factor 21 (TCF21) are associated with cell communication and were identified to be induced by silencing *PYCR1*. Furthermore, HRG inhibits the tumor growth by facilitating clearance of dying tumor cells 53, 54; whereas NR1H4 deficiency has been shown to promote cell proliferation, inflammation, and tumorigenesis in the intestine 55, 56. TCF21 is a specific class II basic helix-loop-helix transcription factor, which functions as a tumor suppressor, likely by inactivating the PI3K/AKT signaling pathway 57, 58.

A previous study has also suggested that p38 MAPK pathways may be activated by PYCR1 and by increased expression of its downstream targets that regulate cell proliferation and metastasis 59. Another study found that the IRS1/JNK signaling pathway was significantly altered after *PYCR1* silencing. IRS1 is a cytoplasmic substrate of the insulin receptor and insulin-like growth factor 1 receptor and plays prominent roles in human malignancies. Phosphorylation of IRS1 leads to the recruitment of downstream effectors and to the activation of the MAPK cascade, which promotes the activation of the PI3K cascade 23. In this study, we identified specific genes involved in MAPK pathways, such as apolipoprotein A1 (APOA1) and Erb-B2 receptor tyrosine kinase 4 (ERBB4), which were differentially expressed following *PYCR1* silencing. Silencing of PYCR1 could interfere cell proliferation through the alteration of IRS1/JNK signaling pathway which subsequently inhibited MAPK pathways.

Other studies have reported that APOA1 may inhibit the formation of tumor vessels and induce an antitumor immune microenvironment, thereby preventing tumor progression 60, 61. Moreover, APOA1-induced apoptosis has been found to be closely related to the inhibition of the MAPK pathway 62. It has also been reported that ERBB4 can enhance the proangiogenic potential via activation of the PI3K/AKT and MAPK/ERK pathways 63. Furthermore, silencing of *PYCR1* has been associated with the inhibition of proliferation and invasiveness of breast cancer cell lines via activation of the AKT/ERK signaling pathway 35. Additionally, caveolin (CAV)-1 and CAV2, which are involved in cell proliferation pathways, have been shown to be necessary for the control of E2-dependent cell growth 64. Thus, downregulation of CAV1 results in increased cell proliferation; whereas downregulation of CAV2 promotes the growth of tumor cells 65, 66.

Many other functional categories were found to be associated with the DEGs identified in *PYCR1*-silenced cells. In particular, glycosylation, cell differentiation, homeostasis, cell adhesion, neuron/nervous system, cell component, and immune stimulus categories were identified. Taken together these results suggest that downregulation of PYCR1 induces the activation of these functional pathways, which may subsequently influence the development of HCC.

The results of our molecular docking analysis suggest that PYCR1 binds to its interacting proteins via binding hotspots. In the MAPK cascade, the DUSP9-PYCR1 complex exhibited the lowest E_total_ value (-906.94 kcal·mol^-1^) compared with those of two other complexes (APOA1-PYCR1 and GRIK2-PYCR1). Further, complexes of PYCR1 with proteins that are most strongly related to cell migration and cell communication exhibited the lowest E_total_ values, namely, SERPINE1-PYCR1 (-1,889.79 kcal·mol^-1^) and NR1H4-PYCR1 (-1,659.98 kcal·mol^-1^). Previous research has suggested that lower E_total_ values are associated with easier and stronger binding between a ligand and a receptor 31. Therefore, our results suggest that functional binding between PYCR1 and DUSP9, SERPINE1, and NR1H4 may mediate the MAPK cascade, cell migration, and cell communication, respectively.

Analysis of hotspots revealed that several amino acid residues in DUSP9 and SERPINE1 exclusively contributed to PYCR1 binding, whereas no hotspots were identified in other protein-protein complexes involved in the MAPK cascade, cell migration, and cell-cell communication. Moreover, several hotspots in the CGA-PYCR1 complex were found to be involved in cell proliferation and growth. These results suggest that the CGA-PYCR1 complex is multifunctional. Interestingly, all of the identified PYCR1 hotspots included Arg, which may imply that Arg is vital for the binding between PYCR1 and its interacting proteins. It has been reported that the depletion of Arg may suppress the proliferation of HCC cells because reduced Arg levels would interfere with intracellular glutamine metabolism and inhibit the synthesis of proteins and thymidine 67, 68. Since our results indicate that Arg may play an important role in the binding between PYCR1 and its interacting proteins, Arg may serve as a potential target for HCC treatment.

Various studies have demonstrated that noncoding RNAs participate in HCC development 69. As a family of small and evolutionarily conserved noncoding RNAs, miRNAs are capable of regulating physiological and pathological processes by inhibiting target mRNA translation or promoting mRNA degradation 70. Our *in silico* prediction revealed that miR-2355-5p might be an upstream regulator for *PYCR1* mRNA. Importantly, when PYCR1 levels were upregulated, miR-2355-5p was downregulated in HCC tissues. These results indicated that miR-2355-5p could serve as a negative regulator of PYCR1 expression, and low miR-2355-5p expression could promote the accumulation of PYCR1 in HCC tissues. Furthermore, miR-2355-5p has been reported to be a master regulator of target genes in other diseases, including chondrosarcoma and intervertebral disc degeneration 71, 72. However, the specific mechanism underlying downregulation of miR-2355-5p in HCC remains unclear.

In summary, our results suggest a role of PYCR1 in inducing the development of HCC. PYCR1 was found to be upregulated in HCC tissues compared to paired adjacent nontumor tissues. Using a TCGA dataset, we found that patients with low PYCR1 expression exhibited a higher OS rate than in patients with high PYCR1 expression. Meanwhile, higher PYCR1 expression was observed in females than in males and correlated with elevated AFP levels, higher clinical staging (stages III and IV), and a younger age (< 45 years old). In Chinese patients, two features, namely, higher clinical staging (stages III and IV) and a younger age (<45 years old), were related to higher PYCR1 expression. When *PYCR1* expression was silenced, the proliferation, migration, invasion, metastasis, and EMT, as well as the tumorigenic capacity, were significantly inhibited in HCC cells. The mechanisms responsible for these effects involved many proteins, which were associated with many functional categories and signaling pathways. Analysis of protein-protein interactions indicated that specific hotspots might facilitate the formation of HCC-specific PYCR1 complexes and promote the development of HCC. We therefore propose that PYCR1 may be an effective novel target for the development of diagnostic or therapeutic options for HCC.

## Supplementary Material

Supplementary figures and tables.

## Figures and Tables

**Table 1:** Correlation between PYCR1 expression and clinicopathological characteristics in HCC patients from the TCGA datasets (n=363)

Clinical features	Case	PYCR1 level (RSEM; mean ±SD)	*p* value
**Sample**			
LIHC	363	7.8001±2.7867	*p*<0.05*
Adjacent tissues	50	6.7891±0.6630	
Age at diagnosis (years)			
≤45	48	8.7148±2.5031	*p*<0.05*
>45	314	7.6514±2.8046	
Unknown	1		
**Gender**			
Female	117	8.3916±2.4315	*p*<0.05*
Male	246	7.5188±2.9032	
**The AFP in serum**			
≤20 ng/ml	143	6.8501±2.7379	*p*<0.05*
>20 ng/ml	129	8.4698±2.7093	
Unknown	91		
**Pathologic stage**			
I-II	251	7.4905±2.8598	*p*<0.05*
III-IV	88	8.6980±2.4119	
Unknown	24		
**Child-Pugh classification**			
A	213	7.4002±2.8886	p>0.05
B	21	8.1762±3.1039	
C	1		
Unknown	128		

TCGA: The Cancer Genome Atlas; LIHC: Liver Hepatocellular Carcinoma; HCC: hepatocellular carcinoma; RSEM: RNA-Seq by Expectation-Maximization; PYCR1: Pyrroline-5-carboxylate reductase 1; AFP: Alpha Fetoprotein; SD: standard deviation. *p < 0.05.

**Table 2:** Correlation between the factors and clinicopathological characteristics in hepatocellular carcinoma (n = 106)

Clinical features	Case number	PYCR1		*P* value
		Low/Neg (*n =* 60)	High (*n =* 46)	
**Age (years)**				
≤45	47	21	26	0.032<0.05*
>45	59	39	20	
**Gender**				
Male	84	45	39	0.239>0.05
Female	22	15	7	
**Smoking**				
Yes	44	21	23	0.164>0.05
No	62	39	23	
**Drinking**				
Yes	44	25	19	1.000>0.05
No	62	35	27	
**HBsAg infection**				
Yes	83	50	33	0.163>0.05
No	23	10	13	
**AFP (ng/mL)**				
≤20	32	19	13	0.832>0.05
>20	74	41	33	
**ALT (U/L)**				
≤40	61	35	26	1.000>0.05
>40	45	25	20	
**AST (U/L)**				
≤40	53	27	26	0.327>0.05
>40	53	33	20	
**Clinical staging**				
I + II	58	39	19	0.019<0.05*
III + IV	48	21	27	
**Tumor size (cm)**				
≤5	43	25	18	0.843>0.05
>5	63	35	28	
**Metastasis**				
Yes	15	6	9	0.174>0.05
No	91	54	37	
**Invasion (DHCC)**				
Yes	50	27	23	1.000>0.05
No	44	24	20	
Unknown	12	9	3	
**Lack of P53**				
Yes	70	37	33	0.274>0.05
No	29	19	10	
Unknown	7	4	3	
**Sample**				
HCC	106	60	46	0.021<0.05*
Adjacent tissue	106	77	29	

AFP: Alpha Fetoprotein; DHCC: diffuse hepatocellular carcinoma; Neg: negative; NS: no significance; *: p < 0.05.

**Table 3:** Molecular docking total energy values for PYCR1 and its interactor proteins with hot spot analysis

Target proteins	Name of PDB files	Positions	E_total_ kcal·mol^-1^	Hot spots of PYCR1	Hot spots of target protein
APOA1	3K2S	25-267	-436.7	/	/
GRIK2	3QXM	429-544, 667-806	-513.79	/	/
ARHGDIB	1DS6	23-199	-820.41	/	/
DUSP9	2HXP	201-345	-906.94	ARG200(A), GLU221(B)	HIS251(G), GLU278(G), GLN282(G)
CGA	1HD4	25-116	-1488.78	ASP165(A), ARG264(A), ARG266(A), GLU267(A), GLN269(A), SER270(A), MET271(A), ILE263(C)	LEU41(G), ARG42(G), SER43(G), VAL61(G), LYS63(G), TYR65(G), TYR88(G), TYR89(G), SER92(G)
RBP4	5NU7	19-200	-1656.65	/	/
NR1H4	4QE6	258-486	-1659.98	/	/
SERPINE1	1C5G	1-402	-1889.79	SER43(B), ARG46(B)	LEU211(G), THR228(G), ASN229(G)
