# Supplementary material for: Deciphering the effects of PYCR1 on cell function and its associated mechanism in hepatocellular carcinoma
Source: Int J Biol Sci. 2021 Jun 1;17(9):2223–39. doi: 10.7150/ijbs.58026 (PMC8241733; doi:10.7150/ijbs.58026)
Supplement: Supplementary file 1 — Supplementary figures and tables. [file ijbsv17p2223s1.pdf]

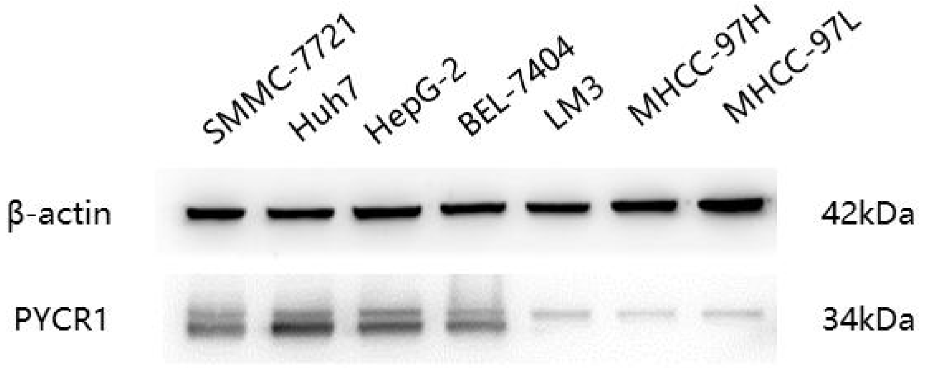

Figure S1. Western blot analysis was used to detect PYCR1 expression in HCC cell lines.

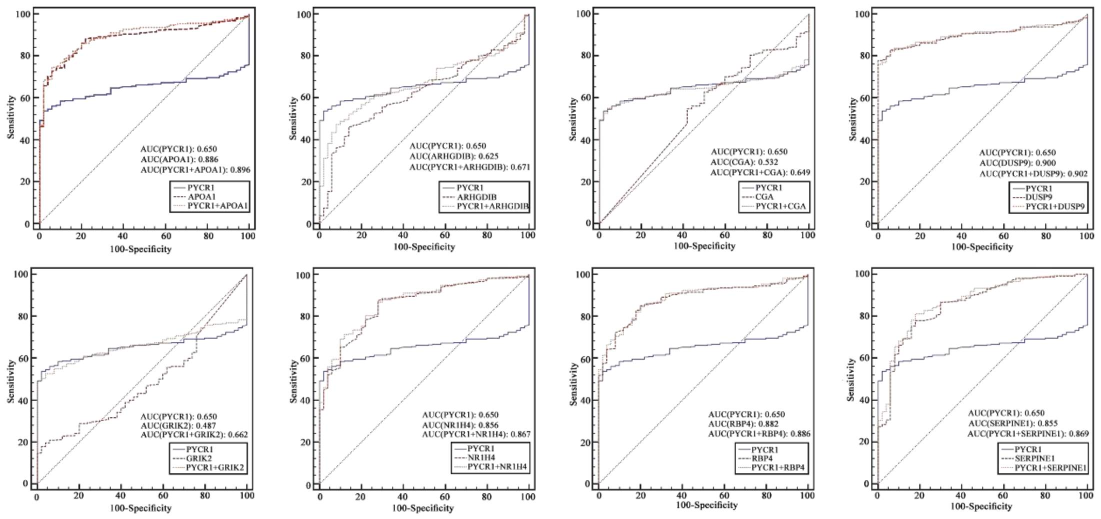

Figure 2S. ROC curves for PYCR1 combining with its interacting proteins.

Table S1. Sequences of specific primers used for qRT-PCR analysis (5′-3′ orientation).

Gene	Primer
Actin-Forward	TGGCACCCAGCACAATGAA
Actin-Reverse	CTAAGTCATAGTCCGCCTAGAAGCA
PYCR1-Forward	GGTCTCCGGACAGCATGAG
PYCR1-Reverse	TGTGGGGTGTCAACTTCACC
CGA-Forward	CTTCGGATCCACAGTCAACC
CGA-Reverse	GAGCGGAATGGAGAACATGC
DIO3-Forward	GTGATGTCGTGGATGGGGAG
DIO3-Reverse	GCTCGTGGTCAAGACCAGTT
TENM1-Forward	TGTCATTTGACAGCAGGCGT
TENM1-Reverse	CTTCTCAGGCAGGAAAGGGG
CAV2-Forward	GCTGTCTGCACATCTGGATT
CAV2-Reverse	CGTCCTACGCTCGTACACAA
CAV1-Forward	GGGGTGTTCCGAGAGAGGTA
CAV1-Reverse	CGGTGTAGAGATGTCCCTGCG
ARHGDIB-Forward	AGCTTTGACGCTAGCCAGG
ARHGDIB-Reverse	TGGCACAAAGACTCCTGAACA
SERPINE1-Forward	CCTGGTTCTGCCCAAGTTCT
SERPINE1-Reverse	CCACTCTCGTTCACCTCGAT
DUSP9-Forward	CCGTCCTAATCAACGTGCCT
DUSP9-Reverse	CAACCACCTGGAGAGGGAAC
TMEFF2-Forward	ATACGTTGTTCCCGGTCCTG
TMEFF2-Reverse	GGGGCATTTCCTTGTGATGC
GRIK2-Forward	CATGCAGCAAGGTTCTGAGC
GRIK2-Reverse	CACTGTCAGAAAGGCGGCTA
HRG-Forward	TGGACTTCTCTGTGCGGAAC
HRG-Reverse	TTCTCATGTTCCTGAGGGTCG
NR1H4-Forward	GGGAATGTTGGCTGAATGCTT
NR1H4-Reverse	TCGCAAGTCACGACCTTCAC
MOXD1-Forward	TCTACAGACCAGTCACGACCT
MOXD1-Reverse	CCTTGAATCGACCACTCAGCA
APOA1-Forward	CAGCGGCAGAGACTATGTGT
APOA1-Reverse	GCTGTCCCAGTTGTCAAGGA
RBP4-Forward	TGGCACGAGTGCAGGGTAAC
RBP4-Reverse	TCTTGCCCAGGAATCCGC
GPC3-Forward	CAAGGCTGTATGGCAGGTGT
GPC3-Reverse	TCTTCTCAGTTTCAGTGGTGGTC
FGB-Forward	CAAGAAGAGAGAAGAGGCTTTGC
FGB-Reverse	GAAGAGGAGGTCTGGGAAACAG
SPTSSB-Forward	ACCTAAGCCGCAGGGAGATA
SPTSSB-Reverse	GCAGTAAGTTTGTCCTAAGAAAGT
TCF21-Forward	CAACCTGACGTGGCCCTTTATG
TCF21-Reverse	GGAAGCAGAGACAGAGAGCAC

SPP1-Forward	GCTTTACAACAAATACCCAGATGC
SPP1-Reverse	GGACTTACTTGGAAGGGTCTGTG
H19-Forward	GCTTGGAAATGAATATGCTGC
H19-Reverse	TCCTCTAGCTTCACCTTCC
U6- Forward	CGCAAGGATGACACGCAAATTC
miR-2355-5p-Forward	ATCCCCAGATACAATGGACAA

Table 2S. Molecular docking results of positive controls with PYCR1.

**Target proteins**	**Confirming experiments**	**Name of PDB files**	**Position**	E_{total} **kcal·mol**$^{-1}$
PARK7	CO-IP	2RK3	1-189	-1139.12
FERMT2	CO-IP; MS	4F7H	328-499	-1219.99
RRM2B	CO-IP; MS	4DJN	13-322	-1566.48
SIRT3	CO-IP	4BN4	116-399	-1635.58
STAT3	CO-IP	6NJS	127-688	-1739.72

PARK7: Parkinson disease protein 7; FERMT2: Fermitin family homolog 2; RRM2B: Ribonucleoside-diphosphate reductase subunit M2 B; SIRT3: NAD-dependent protein deacetylase sirtuin-3; STAT3: Signal transducer and activator of transcription 3; CO-IP: co-immunoprecipitation; MS: mass spectrometry

Table 3S: Sensitivity and specificity to diagnose hepatocellular carcinoma with PYCR1 and its interacting proteins.

Model	AUC (95%CI)	Base on TCGA analysis	
		Sensitivity (%)	Specificity (%)
PYCR1	0.650 (0.602 - 0.696)	53.7	98.0
PYCR1 + APOA1	0.896 (0.823 - 0.924)	74.7	74.0
PYCR1 + ARHGDIB	0.671 (0.624 - 0.716)	47.4	92.0
PYCR1 + CGA	0.649 (0.601 – 0.695)	55.1	96.0
PYCR1 + DUSP9	0.902 (0.869 – 0.929)	82.4	94.0
PYCR1 + GRIK2	0.662 (0.614 – 0.708)	49.0	100
PYCR1 + NR1H4	0.867 (0.830 – 0.898)	71.3	88.0
PYCR1 + RBP4	0.886 (0.851 - 0.915)	84.6	80.0
PYCR1 + SERPINE1	0.869 (0.833 – 0.900)	81.3	82.0

Table 4S. Predicted miRNAs targeting PYCR1 from each database.

Online database	Species	Predicted miRNA ID
miRDB	Homo sapiens	hsa-miR-1253
	Homo sapiens	hsa-miR-323a-5p
	Homo sapiens	hsa-miR-378a-5p
	Homo sapiens	hsa-miR-4270
	Homo sapiens	hsa-miR-4287
	Homo sapiens	hsa-miR-4290
	Homo sapiens	hsa-miR-4436a
	Homo sapiens	hsa-miR-4441
	Homo sapiens	hsa-miR-4469
	Homo sapiens	hsa-miR-4492
	Homo sapiens	hsa-miR-4504
	Homo sapiens	hsa-miR-4520-5p
	Homo sapiens	hsa-miR-4747-5p
	Homo sapiens	hsa-miR-5000-3p
	Homo sapiens	hsa-miR-5196-5p
	Homo sapiens	hsa-miR-520g-5p
	Homo sapiens	hsa-miR-548a-3p
	Homo sapiens	hsa-miR-548ar-3p
	Homo sapiens	hsa-miR-548az-3p
	Homo sapiens	hsa-miR-548bc
	Homo sapiens	hsa-miR-548e-3p
	Homo sapiens	hsa-miR-548f-3p
	Homo sapiens	hsa-miR-602
	Homo sapiens	hsa-miR-6081
	Homo sapiens	hsa-miR-6764-5p
	Homo sapiens	hsa-miR-6867-3p
	Homo sapiens	hsa-miR-7113-3p
	Homo sapiens	hsa-miR-7150
TargetScan	Homo sapiens	hsa-miR-129-3p
	Homo sapiens	hsa-miR-199-5p
	Homo sapiens	hsa-miR-133a-3p.1
	Homo sapiens	hsa-miR-802
	Homo sapiens	hsa-miR-383-5p.1
	Homo sapiens	hsa-miR-133a-3p.2
	Homo sapiens	hsa-miR-140-3p.2
	Homo sapiens	hsa-miR-192-5p
	Homo sapiens	hsa-miR-128-3p
	Homo sapiens	hsa-miR-10-5p
	Homo sapiens	hsa-miR-9-5p

Homo sapiens	hsa-miR-150-5p
Homo sapiens	hsa-miR-125-5p
Homo sapiens	hsa-miR-29-3p
Homo sapiens	hsa-miR-124-3p.1
Homo sapiens	hsa-miR-137
Homo sapiens	hsa-miR-27-3p
Homo sapiens	hsa-miR-219-5p
Homo sapiens	hsa-miR-124-3p.2
Homo sapiens	hsa-miR-145-5p
Homo sapiens	hsa-miR-216b-5p
Homo sapiens	hsa-miR-33-5p
Homo sapiens	hsa-miR-489-3p
Homo sapiens	hsa-miR-1-3p
Homo sapiens	hsa-miR-423-5p
Homo sapiens	hsa-miR-328-3p
Homo sapiens	hsa-miR-331-3p
Homo sapiens	hsa-miR-342-3p
Homo sapiens	hsa-miR-504-5p.1
Homo sapiens	hsa-miR-1197
Homo sapiens	hsa-miR-491-5p
Homo sapiens	hsa-miR-378-3p
Homo sapiens	hsa-miR-326
Homo sapiens	hsa-miR-532-3p
Homo sapiens	hsa-miR-3064-5p
Homo sapiens	hsa-miR-299-3p
Homo sapiens	hsa-miR-877-5p
Homo sapiens	hsa-miR-670-3p
Homo sapiens	hsa-miR-874-3p
Homo sapiens	hsa-miR-136-5p
Homo sapiens	hsa-miR-488-3p
Homo sapiens	hsa-miR-377-3p
Homo sapiens	hsa-miR-339-5p
Homo sapiens	hsa-miR-873-5p.1
Homo sapiens	hsa-miR-340-5p
Homo sapiens	hsa-miR-543
Homo sapiens	hsa-miR-5194
Homo sapiens	hsa-miR-378g
Homo sapiens	hsa-miR-3150a-3p
Homo sapiens	hsa-miR-5000-3p
Homo sapiens	hsa-miR-4640-5p
Homo sapiens	hsa-miR-3173-5p
Homo sapiens	hsa-miR-2114-3p

Homo sapiens	hsa-miR-4739
Homo sapiens	hsa-miR-2355-5p
Homo sapiens	hsa-miR-588
Homo sapiens	hsa-miR-511-3p
Homo sapiens	hsa-miR-3690
Homo sapiens	hsa-miR-4640-3p
Homo sapiens	hsa-miR-4731-5p
Homo sapiens	hsa-miR-766-5p
Homo sapiens	hsa-miR-654-5p
Homo sapiens	hsa-miR-1266-3p
Homo sapiens	hsa-miR-214-3p
Homo sapiens	hsa-miR-345-3p
Homo sapiens	hsa-miR-670-5p
Homo sapiens	hsa-miR-545-5p
Homo sapiens	hsa-miR-605-3p
Homo sapiens	hsa-miR-3194-5p
Homo sapiens	hsa-miR-450b-5p
Homo sapiens	hsa-miR-508-3p
Homo sapiens	hsa-miR-510-5p
Homo sapiens	hsa-miR-642b-5p
Homo sapiens	hsa-miR-525-5p
Homo sapiens	hsa-miR-3605-5p
Homo sapiens	hsa-miR-3179
Homo sapiens	hsa-miR-3918
Homo sapiens	hsa-miR-518d-5p
Homo sapiens	hsa-miR-4766-5p
Homo sapiens	hsa-miR-574-5p
Homo sapiens	hsa-miR-5691
Homo sapiens	hsa-miR-4428
Homo sapiens	hsa-miR-873-3p
Homo sapiens	hsa-miR-660-5p
Homo sapiens	hsa-miR-513a-5p
Homo sapiens	hsa-miR-2114-5p
Homo sapiens	hsa-miR-675-3p
Homo sapiens	hsa-miR-625-5p
Homo sapiens	hsa-miR-1252-5p
Homo sapiens	hsa-miR-4761-3p
Homo sapiens	hsa-miR-516b-5p
Homo sapiens	hsa-miR-324-3p
Homo sapiens	hsa-miR-345-5p
Homo sapiens	hsa-miR-1343-3p
Homo sapiens	hsa-miR-370-3p

	Homo sapiens	hsa-miR-642-3p
	Homo sapiens	hsa-miR-642a-5p
	Homo sapiens	hsa-miR-2116-3p
	Homo sapiens	hsa-miR-197-3p
	Homo sapiens	hsa-miR-3127-5p
	Homo sapiens	hsa-miR-3163
	Homo sapiens	hsa-miR-133b
	Homo sapiens	hsa-miR-215-5p
	Homo sapiens	hsa-miR-506-3p
	Homo sapiens	hsa-miR-206
	Homo sapiens	hsa-miR-3619-5p
	Homo sapiens	hsa-miR-519-5p
	Homo sapiens	hsa-miR-1913
	Homo sapiens	hsa-miR-34a-5p
	Homo sapiens	hsa-miR-92a-1-5p
	Homo sapiens	hsa-miR-129-1-3p
	Homo sapiens	hsa-miR-129-2-3p
	Homo sapiens	hsa-miR-377-5p
	Homo sapiens	hsa-miR-920
	Homo sapiens	hsa-miR-940
	Homo sapiens	hsa-miR-1233-3p
	Homo sapiens	hsa-miR-1253
	Homo sapiens	hsa-miR-2110
	Homo sapiens	hsa-miR-3150a-3p
	Homo sapiens	hsa-miR-3173-3p
	Homo sapiens	hsa-miR-4300
	Homo sapiens	hsa-miR-2355-5p
miRNet/miRTarBase	Homo sapiens	hsa-miR-4271
	Homo sapiens	hsa-miR-3679-3p
	Homo sapiens	hsa-miR-3919
	Homo sapiens	hsa-miR-4468
	Homo sapiens	hsa-miR-4690-3p
	Homo sapiens	hsa-miR-4725-3p
	Homo sapiens	hsa-miR-5591-5p
	Homo sapiens	hsa-miR-5685
	Homo sapiens	hsa-miR-6081
	Homo sapiens	hsa-miR-6086
	Homo sapiens	hsa-miR-6090
	Homo sapiens	hsa-miR-655-5p
	Homo sapiens	hsa-miR-3912-5p
	Homo sapiens	hsa-miR-6726-5p
	Homo sapiens	hsa-miR-6763-5p

Homo sapiens	hsa-miR-6793-3p
Homo sapiens	hsa-miR-6810-5p
Homo sapiens	hsa-miR-6814-5p
Homo sapiens	hsa-miR-6827-5p
Homo sapiens	hsa-miR-6780b-5p
Homo sapiens	hsa-miR-6857-5p
Homo sapiens	hsa-miR-6891-5p
Homo sapiens	hsa-miR-450a-2-3p
Homo sapiens	hsa-let-7e-5p
